# Supplementary material for: Sequence-independent, site-specific incorporation of chemical modifications to generate light-activated plasmids
Source: Chem Sci. 2023 Oct 13;14(44):12693–706. doi: 10.1039/d3sc02761a (PMC10646958; doi:10.1039/d3sc02761a)
Supplement: SC-014-D3SC02761A-s001 [file SC-014-D3SC02761A-s001.pdf]

Electronic Supplementary Material (ESI) for Chemical Science.
This journal is © The Royal Society of Chemistry 2023

Supplementary Information

Sequence-independent, site-specific incorporation of chemical modifications to generate light-activated plasmids

Authors: Khoa Chung,[1] Michael J. Booth[1,2*]

Affiliations:

[1]Department of Chemistry, University of Oxford, Mansfield Road, OX1 3TA, Oxford, UK.

[2]Department of Chemistry, University College London, 20 Gordon Street, London WC1H 0AJ, U.K.

[*]Correspondence: m.j.booth@ucl.ac.uk

Contents

DNA Sequences 3

Supplementary Table 1. Primers used to assess dinucleotides at the ligation junction 3

Supplementary Table 2. mV_nicks and mNG_BbvCI plasmid sequences 3

Supplementary Table 3. Primers used for PCR, RT-qPCR and NEEL 7

Supplementary Table 4. Primers used to synthesise the backbones and inserts for homologous recombinations 8

Supplementary Table 5. Backbone and insert combinations for homologous recombinations 8

Supplementary Table 6. HPLC Purification of the PCB oligos 8

Supplementary Data 9

SI Figure 1. Different Kunkel methods were employed to resynthesise the plasmid 9

SI Figure 2. NEEL with all possible dinucleotide combinations at the ligation junction 10

SI Figure 3. DpnI removal of unreacted plasmids 11

SI Figure 4. Confirming synthesis of the T7 PCB oligo 12

SI Figure 5. PCB oligo was unaffected by annealing conditions 13

SI Figure 6. Comparison between MVS and WTS LA-plasmids 14

SI Figure 7. Negative controls without amino modifications 15

SI Figure 8. CFPS of T7 NEEL plasmids including the native plasmid. 16

SI Figure 9. Confirming synthesis of the CMV PCB oligo A. 17

SI Figure 10. Cell viability assay with optimised UV settings for transfection 18

SI Figure 11. CMV LA-plasmids and their uncaging 19

SI Figure 12. RT-qPCR and fluorescence measurements of CMV NEEL plasmids, including the native plasmid 20

SI Figure 13. GAPDH transcription levels after transfection with modified plasmids 21

References 22

DNA Sequences

Supplementary Table 1. Primers used to assess dinucleotides at the ligation junction

The primers followed the format X_Y_FOR or X_Y_phos where X represents the nucleotide at the 3′-end and Y represents the nucleotide at the 5′-end of the ligation junction. 'phos' primers were already 5′ phosphorylated.

Name	Sequence
C_C_FOR	CTGAAGCTCATCTGCACC
G_T_FOR	TCCAGGAGCGCACCATCTTC
T_C_FOR	CAAGGACGACGGCAACTAC
G_C_FOR	CATCGAGCTGAAGGGCATC
C_T_FOR	TTCAAGGAGGACGGCAACATCC
G_G_FOR	GGAATTCTCGAGTAAGGTTAACC
T_A_FOR	ATATCCGGAAGCTTGGCACTGG
G_A_FOR	AGCGGTATCAGCTCACTC
C_A_FOR	AAGTCAGAGGTGGCGAAACCC
A_A_FOR	AGCGTGGCGCTTTCTCATAGC
T_G_phos	GTAGGTCGTTCGCTCCAAGC
A_G_FOR	GTAAACTTGGTCTGACAGTTACC
A_T_FOR	TCTCAGCGATCTGTCTATTTCG
T_T_FOR	TCATCCATAGTTGCCTGAC
C_G_phos	GAGTTGCTCTTGCCCGGCGTC
A_C_phos	CACGGAAATGTTGAATACTC

Supplementary Table 2. mV_nicks and mNG_BbvCI plasmid sequences

Plasmid	Sequence
mV_nicks	GCTAGTGGTGCTAGCCCCGCGAAATTAATACGACTCACTATAGG GTCTAGAAATAATTTTGTTTAACTTTAAGAAGGAGGTATACATA TGGTGAGCAAGGGCGAGGAGCTGTTCACCGGGGTGGTGCCCAT CCTGGTCGAGCTGGACGGCGACGTAAACGGCCACAAGTTCAGC GTGTCCGGCGAGGGCGAGGGCGATGCCACCTACGGCAAGCTGA CCCTGAAGCTCATCTGCACCACCGGCAAGCTGCCCGTGCCCTGG CCCACCCTCGTGACCACCCTCGGCTACGGCCTGCAGTGCTTCGC CCGCTACCCCGACCACATGAAGCAGCACGACTTCTTCAAGTCCG CCATGCCCGAAGGCTACGTCCAGGAGCGCACCATCTTCTTCAAG GACGACGGCAACTACAAGACCCGCGCCGAGGTGAAGTTCGAGG GCGACACCCTGGTGAACCGCATCGAGCTGAAGGGCATCGACTT CAAGGAGGACGGCAACATCCTGGGGCACAAGCTGGAGTACAAC TACAACAGCCACAACGTCTATATCACCGCCGACAAGCAGAAGA ACGGCATCAAGGCCAACTTCAAGATCCGCCACAACATCGAGGA CGGCGGCGTGCAGCTCGCCGACCACTACCAGCAGAACACCCCC ATCGGCGACGGCCCCGTGCTGCTGCCCGACAACCACTACCTGAG CTACCAGTCCAAGCTGAGCAAAGACCCCAACGAGAAGCGCGAT CACATGGTCCTGCTGGAGTTCGTGACCGCCGCCGGGATCACTCT CGGCATGGACGAGCTGTACAAGTAATGACCTCAGCGGATCCGC TCTTCCGGGAATTCTCGAGTAAGGTTAACCTGCAGGAGGCCTTT AATTAAGGTGGTGCGGCCGCGCTAGCGGTCCCGGGGGATCGAT CCGGCTGCTAACAAAGCCCGAAAGGAAGCTGAGTTGGCTGCTG CCACCGCTGAGCAATAACTAGCATAACCCCTTGGGGCCTCTAAA

	CGGGTCTTGAGGGGTTTTTGCTGAAAGGAGGAACTATATCCGG AAGCTTGGCACTGGCCGACCGGGGTCGAGCACTGACTCGCTGC GCTCGGTCGTTCGGCTGCGGCGAGCGGTATCAGCTCACTCAAAG GCGGTAATACGGTTATCCACAGAATCAGGGGATAACGCAGGAA AGAACATGTGAGCAAAAGGCCAGCAAAAGGCCAGGAACCGTA AAAAGGCCGCGTTGCTGGCGTTTTTCCATAGGCTCCGCCCCCCT GACGAGCATCACAAAAATCGACGCTCAAGTCAGAGGTGGCGAA ACCCGACAGGACTATAAAGATACCAGGCGTTTCCCCCTGGAAG CTCCCTCGTGCGCTCTCCTGTTCCGACCCTGCCGCTTACCGGATA CCTGTCCGCCTTTCTCCCTTCGGGAAGCGTGGCGCTTTCTCATAG CTCACGCTGTAGGTATCTCAGTTCGGTGTAGGTCGTTCGCTCCA AGCTGGGCTGTGTGCACGAACCCCCCGTTCAGCCCGACCGCTGC GCCTTATCCGGTAACTATCGTCTTGAGTCCAACCCGCTAAGACA CGACTTATCGCCACTGGCAGCAGCCACTGGTAACAGGATTAGCA GAGCGAGGTATGTAGGCGGTGCTACAGAGTTCTTGAAGTGGTG GCCTAACTACGGCTACACTAGAAGAACAGTATTTGGTATCTGCG CTCTGCTGAAGCCAGTTACCTTCGGAAAAAGAGTTGGTAGCTCT TGATCCGGCAAACAAACCACCGCTGGTAGCGGTGGTTTTTTTGT TTGCAAGCAGCAGATTACGCGCAGAAAAAAAGGATCTCAAGAA GATCCTTTGATCTTTTCTACGGGGTCTGACGCTCAGTGGAACGA AAACTCACAGATCCGGGATTTTGGTCATGAGATTATCAAAAAGG ATCTTCACCTAGATCCTTTTAAATTAAAAATGAAGTTTTAAATC AATCTAAAGTATATATGAGTAAACTTGGTCTGACAGTTACCAAT GCTTAATCAGTGAGGCACCTATCTCAGCGATCTGTCTATTTCGTT CATCCATAGTTGCCTGACTCCCCGTCGTGTAGATAACTACGATA CGGGAGGGCTTACCATCTGGCCCCAGTGCTGCAATGATACCGCG AGACCCACGCTCACCGGCTCCAGATTTATCAGCAATAAACCAGC CAGCCGGAAGGGCCGAGCGCAGAAGTGGTCCTGCAACTTTATC CGCCTCCATCCAGTCTATTAATTGTTGCCGGGAAGCTAGAGTAA GTAGTTCGCCAGTTAATAGTTTGCGCAACGTTGTTGCCATTGCT ACAGGCATCGTGGTGTCACGCTCGTCGTTTGGTATGGCTTCATT CAGCTCCGGTTCCCAACGATCAAGGCGAGTTACATGATCCCCCA TGTTGTGCAAAAAAGCGGTTAGCTCCTTCGGTCCTCCGATCGTT GTCAGAAGTAAGTTGGCCGCAGTGTTATCACTCATGGTTATGGC AGCACTGCATAATTCTCTTACTGTCATGCCATCCGTAAGATGCTT TTCTGTGACTGGTGAGTACTCAACCAAGTCATTCTGAGAATAGT GTATGCGGCGACCGAGTTGCTCTTGCCCGGCGTCAATACGGGAT AATACCGCGCCACATAGCAGAACTTTAAAAGTGCTCATCATTGG AAAACGTTCTTCGGGGCGAAAACTCTCAAGGATCTTACCGCTGT TGAGATCCAGTTCGATGTAACCCACTCGTGCACCCAACTGATCT TCAGCATCTTTTACTTTCACCAGCGTTTCTGGGTGAGCAAAAAC AGGAAGGCAAAATGCCGCAAAAAAGGGAATAAGGGCGACACG GAAATGTTGAATACTCATACTCTTCCTTTTTCAATATTATTGAAG CATTTATCAGGGTTATTGTCTCATGAGCGGATACATATTTGAAT GTATTTAGAAAAATAAACAAATAGGGGTTCCGCGCACATTTCCC CGAAAAGT
mNG_BbvCI	TCGACCGCCAATTCAATATGGCGTATATGGACTCATGCCAATTC AATATGGTGGATCTGGACCTGTGCCAATTCAATATGGCGTATAT GGACTCGTGCCAATTCAATATGGTGGATCTGGACCCCAGCCAAT TCAATATGGCGGACTTGGCACCATGCCAATTCAATATGGCGGAC

	CTGGCACTGTGCCAACTGGGGAGGGGTCTACTTGGCACGGTGCC<br>AAGTTTGAGGAGGGGTCTTGGCCCTGTGCCAAGTCCGCCATATT<br>GAATTGGCATGGTGCCAATAATGGCGGCCATATTGGCTATATGC<br>CAGGATCAATATATAGGCAATATCCAATATGGCCCTATGCCAAT<br>ATGGCTATTGGCCAGGTTCAATACTATGTATTGGCCCTATGCCA<br>TATAGTATTCCATATATGGGTTTTCCTATTGACGTAGATAGCCCC<br>TCCCAATGGGCGGTCCCATATACCATATATGGGGCTTCCTAATA<br>CCGCCCATAGCCACTCCCCCATTGACGTCAATGGTCTCTATATA<br>TGGTCTTTCCTATTGACGTCATATGGGCGGTCCTATTGACGTATA<br>TGGCGCCTCCCCCATTGACGTCAATTACGGTAAATGGCCCGCCT<br>GGCTCAATGCCCATTGACGTCAATAGGACCACCCACCATTGACG<br>TCAATGGGATGGCTCATTGCCCATTCATATCCGTTCTCACGCCCC<br>CTATTGACGTCAATGACGGTAAATGGCCCACTTGGCAGTACATC<br>AATATCTATTAATAGTAACTTGGCAAGTACATTACTATTGGAAG<br>TACGCCAGGGTACATTGGCAGTACTCCCATTGACGTCAATGGCG<br>GTAAATGGCCCGCGATGGCTGCCAAGTACATCCCCATTGACGTC<br>AATGGGGAGGGGCAATGACGCAAATGGGCGTTCCATTGACGTA<br>AATGGGCGGTAGGCGTGCCTAATGGGAGGTCTATATAAGCAAT<br>GCTCGTTTAGGGAACCGCCATTCTGCCTGGGGACGTCGGAGCAA<br>GCTTGATTTAGGTGACACTATAGACTTGTTCTTTTTGCAGGATCT<br>ACCATGGTGAGCAAGGGCGAGGAGGATAACATGGCCTCTCTCC<br>CAGCGACACATGAGTTACACATCTTTGGCTCCATCAACGGTGTG<br>GACTTTGACATGGTGGGTCAGGGCACCGGCAATCCAAATGATG<br>GTTATGAGGAGTTAAACCTGAAGTCCACCAAGGGTGACCTCCA<br>GTTCTCCCCCTGGATTCTGGTCCCTCATATCGGGTATGGCTTCCA<br>TCAGTACCTGCCCTACCCTGACGGGATGTCGCCTTTCCAGGCCG<br>CCATGGTAGATGGCTCCGGATACCAAGTCCATCGCACAATGCAG<br>TTTGAAGATGGTGCCTCCCTTACTGTTAACTACCGCTACACCTAC<br>GAGGGAAGCCACATCAAAGGAGAGGCCCAGGTGAAGGGGACT<br>GGTTTCCCTGCTGACGGTCCTGTGATGACCAACTCGCTGACCGC<br>TGCGGACTGGTGCAGGTCGAAGAAGACTTACCCCAACGACAAA<br>ACCATCATCAGTACCTTTAAGTGGAGTTACACCACTGGAAATGG<br>CAAGCGCTACCGGAGCACTGCGCGGACCACCTACACCTTTGCCA<br>AGCCAATGGCGGCTAACTATCTGAAGAACCAGCCGATGTACGT<br>GTTCCGTAAGACGGAGCTCAAGCACTCCAAGACCGAGCTCAAC<br>TTCAAGGAGTGGCAAAAGGCCTTTACCGATGTGATGGGCATGG<br>ACGAGCTGTACAAGGGATCTGGATCCCATCGATTCGAATTCAAG<br>GCCTCTCGAGCCTCTAGAACTATAGTGAGTCGTATTACGTAGAT<br>CCAGACATGATAAGATACATTGATGAGTTTGGACAAACCACAA<br>CTAGAATGCAGTGAAAAAAATGCTTTATTTGTGAAATTTGTGAT<br>GCTATTGCTTTATTTGTAACCATTATAAGCTGCAATAAACAAGT<br>TAACAACAACAATTGCATTCATTTTATGTTTCAGGTTCAGGGGG<br>AGGTGTGGGAGGTTTTTTAATTCGCGGCCGCGGCGCCAATGCAT<br>TGGGCCCGGTACCCAGCTTTTGTTCCCTTTAGTGAGGGTTAATTG<br>CGCGCTTGGCGTAATCCCTCAGCATGGTCATAGCTGTTTCCTGT<br>GTGAAATTGTTATCCGCTCACAATTCCACACAACATACGAGCCG<br>GGAGCATAAAGTGTAAAGCCTGGGGTGCCTAATGAGTGAGCTA<br>ACTCACATTAATTGCGTTGCGCTCACTGCCCGCTTTCCAGTCGG<br>GAAACCTGTCGTGCCAGCTGCATTAATGAATCGGCCAACGCGCG<br>GGGAGAGGCGGTTTGCGTATTGGGCGCTCTTCCGCTTCCTCGCT

	CACTGACTCGCTGCGCTCGGTCGTTCGGCTGCGGCGAGCGGTAT
	CAGCTCACTCAAAGGCGGTAATACGGTTATCCACAGAATCAGG
	GGATAACGCAGGAAAGAACATGTGAGCAAAAGGCCAGCAAAA
	GGCCAGGAACCGTAAAAAGGCCGCGTTGCTGGCGTTTTTCCATA
	GGCTCCGCCCCCCTGACGAGCATCACAAAAATCGACGCTCAAGT
	CAGAGGTGGCGAAACCCGACAGGACTATAAAGATACCAGGCGT
	TTCCCCCTGGAAGCTCCCTCGTGCGCTCTCCTGTTCCGACCCTGC
	CGCTTACCGGATACCTGTCCGCCTTTCTCCCTTCGGGAAGCGTG
	GCGCTTTCTCATAGCTCACGCTGTAGGTATCTCAGTTCGGTGTA
	GGTCGTTCGCTCCAAGCTGGGCTGTGTGCACGAACCCCCCGTTC
	AGCCCGACCGCTGCGCCTTATCCGGTAACTATCGTCTTGAGTCC
	AACCCGGTAAGACACGACTTATCGCCACTGGCAGCAGCCACTG
	GTAACAGGATTAGCAGAGCGAGGTATGTAGGCGGTGCTACAGA
	GTTCTTGAAGTGGTGGCCTAACTACGGCTACACTAGAAGAACAG
	TATTTGGTATCTGCGCTCTGCTGAAGCCAGTTACCTTCGGAAAA
	AGAGTTGGTAGCTCTTGATCCGGCAAACAAACCACCGCTGGTAG
	CGGTGGTTTTTTTGTTTGCAAGCAGCAGATTACGCGCAGAAAAA
	AAGGATCTCAAGAAGATCCTTTGATCTTTTCTACGGGGTCTGAC
	GCTCAGTGGAACGAAAACTCACGTTAAGGGATTTTGGTCATGAG
	ATTATCAAAAAGGATCTTCACCTAGATCCTTTTAAATTAAAAAT
	GAAGTTTTAAATCAATCTAAAGTATATATGAGTAAACTTGGTCT
	GACAGTTACCAATGCTTAATCAGTGAGGCACCTATCTCAGCGAT
	CTGTCTATTTCGTTCATCCATAGTTGCCTGACTCCCCGTCGTGTA
	GATAACTACGATACGGGAGGGCTTACCATCTGGCCCCAGTGCTG
	CAATGATACCGCGAGACCCACGCTCACCGGCTCCAGATTTATCA
	GCAATAAACCAGCCAGCCGGAAGGGCCGAGCGCAGAAGTGGTC
	CTGCAACTTTATCCGCCTCCATCCAGTCTATTAATTGTTGCCGGG
	AAGCTAGAGTAAGTAGTTCGCCAGTTAATAGTTTGCGCAACGTT
	GTTGCCATTGCTACAGGCATCGTGGTGTCACGCTCGTCGTTTGG
	TATGGCTTCATTCAGCTCCGGTTCCCAACGATCAAGGCGAGTTA
	CATGATCCCCCATGTTGTGCAAAAAAGCGGTTAGCTCCTTCGGT
	CCTCCGATCGTTGTCAGAAGTAAGTTGGCCGCAGTGTTATCACT
	CATGGTTATGGCAGCACTGCATAATTCTCTTACTGTCATGCCATC
	CGTAAGATGCTTTTCTGTGACTGGTGAGTACTCAACCAAGTCAT
	TCTGAGAATAGTGTATGCGGCGACCGAGTTGCTCTTGCCCGGCG
	TCAATACGGGATAATACCGCGCCACATAGCAGAACTTTAAAAG
	TGCTCATCATTGGAAAACGTTCTTCGGGGCGAAAACTCTCAAGG
	ATCTTACCGCTGTTGAGATCCAGTTCGATGTAACCCACTCGTGC
	ACCCAACTGATCTTCAGCATCTTTTACTTTCACCAGCGTTTCTGG
	GTGAGCAAAAACAGGAAGGCAAAATGCCGCAAAAAAGGGAAT
	AAGGGCGACACGGAAATGTTGAATACTCATACTCTTCCTTTTTC
	AATATTATTGAAGCATTTATCAGGGTTATTGTCTCATGAGCGGA
	TACATATTTGAATGTATTTAGAAAAATAAACAAATAGGGGTTCC
	GCGCACATTTCCCCGAAAAGTGCCACCTAAATTGTAAGCGTTAA
	TATTTTGTTAAAATTCGCGTTAAATTTTTGTTAAATCAGCTCATT
	TTTTAACCAATAGGCCGAAATCGGCAAAATCCCTTATAAATCAA
	AAGAATAGACCGAGATAGGGTTGAGTGTTGTTCCAGTTTGGAAC
	AAGAGTCCACTATTAAAGAACGTGGACTCCAACGTCAAAGGGC
	GAAAAACCGTCTATCAGGGCGATGGCCCACTACGTGAACCATC
	ACCCTAATCAAGTTTTTTGGGGTCGAGGTGCCGTAAAGCACTAA

	ATCGGAACCCTAAAGGGAGCCCCCGATTTAGAGCTTGACGGGG AAAGCCGGCGAACGTGGCGAGAAAGGAAGGGAAGAAAGCGAA AGGAGCGGGCGCTAGGGCGCTGGCAAGTGTAGCGGTCACGCTG CGCGTAACCACCACACCCGCCGCGCTTAATGCGCCGCTACAGGG CGCGTCCCATTCGCCATTCAGGCTGCGCAACTGTTGGGAAGGGC GATCGGTGCGGGCCTCTTCGCTATTACGCCAG

Supplementary Table 3. Primers used for PCR, RT-qPCR and NEEL

Underlined sequences are the T7 promoter and TATA box. Bolded sequences are the upstream and downstream BRE and Inr elements. X represents the amino-C6-dT modifications.

Name	Sequence
mV nick T7T FRW	TACAAGTAATGACCTCAGCGGATCCGCTCTTCCGGGAATTC TCGAGTAAGG
DHFR REV T7S GFP	ACAGCTCCTCGCCCTTGCTCACCATATGTATACCTCCTTCTT AAAGTTAAAC
GFP FRW T7S	CGGCATGGACGAGCTGTACAAGTAATGAGGATCCCGGGAA TTCTCGAGTA
mV nick T7T REV	AATTCCCGGAAGAGCGGATCCGCTGAGGTCATTACTTGTAC AGCTCG
mNG_BbvCI_FOR1	GCTTGGCGTAATCCCTCAGCATGGTCATAGCTG
mNG_BbvCI_REV2	CAGCTATGACCATGCTGAGGGATTACGCCAAGC
mNG_nick_mutagenesis_REV1	CGAGAGGCCTTGAATTCGAATCGATG
mNG_nick_mutagenesis_FOR2	CATCGATTCGAATTCAAGGCCTCTCG
mNG_qPCR_FOR	CGTGTTCCGTAAGACGGAGC
mNG_RT-qPCR_REV	CCTTGTACAGCTCGTCCATGC
GAPDH_FOR_Origene	GTCTCCTCTGACTTCAACAGCG
GAPDH_REV_IDT	TCCACCACCCTGTTGCTGTA
T7_eLong	GAAATTAATACGACTCACTATAGGGTCTAG
T7_7amines	GAAATXAAXACGACXCACXAXAGGGXCXAG
mNG_9amines_46b	**GGXC**XAXAXAAG**CAAXGCX**CGTTTAGGGAACCG**CCATXC** **X**GCCXGG

Supplementary Table 4. Primers used to synthesise the backbones and inserts for homologous recombinations

PCR	DNA template	For primer	Rev Primer
mV_nicks_BB	mVenus_CT	mV nick T7T FRW	DHFR REV T7S GFP
mV_nicks_insert	mVenus_CT	GFP FRW T7S	mV nick T7T REV
mNG_BbvCI_BB	pCS2-mNG-C plasmid	mNG_BbvCI_FOR1	mNG_nick_mutagenesis_REV1
mNG_BbvCI_insert	pCS2-mNG-C plasmid	mNG_BbvCI_REV2	mNG_nick_mutagenesis_FOR2

Supplementary Table 5. Backbone and insert combinations for homologous recombinations

Plasmid	Backbone	Insert
mV_nicks	mV_nicks_BB	mV_nicks_insert
mNG_BbvCI	mNG_BbvCI_BB	mNG_BbvCI_insert

Supplementary Table 6. HPLC Purification of the PCB oligos

Promoter	Number of modifications	Collection period/ mins	Increments/ mins	Fractions pooled
T7	7	24.0-25.5	0.15	5-7
CMV	9	24.0-25.5	0.15	3-5

Supplementary Data

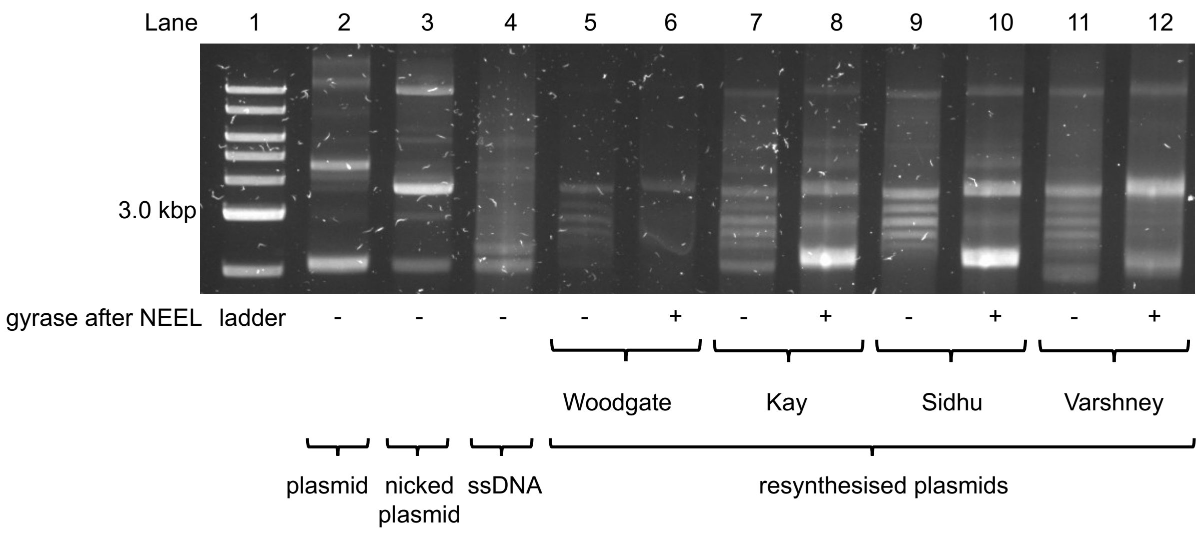

SI Figure 1. Different Kunkel methods were employed to resynthesise the plasmid. Kunkel mutagenesis protocols from the Woodgate,[1] Kay,[2] Sidhu,[3] and Varshney[4] laboratories were used to regenerate the plasmid. After ligation, the different plasmids were reacted with gyrase to test for ligation.

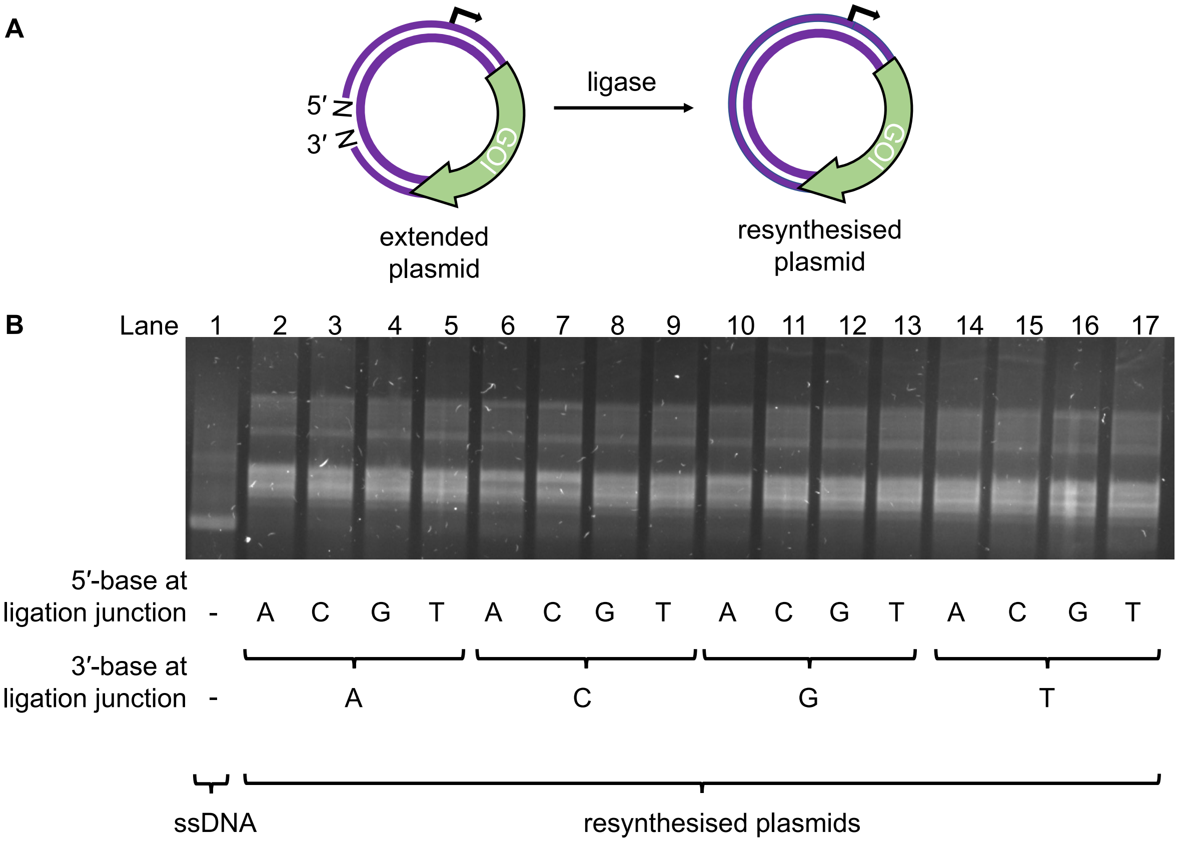

SI Figure 2. NEEL with all possible dinucleotide combinations at the ligation junction. A. Depiction of the reaction in (**B**). **B.** Different primers with varying T_m, GC content, and lengths were used for NEEL. Ligation at the different ligation junctions was equally effective.

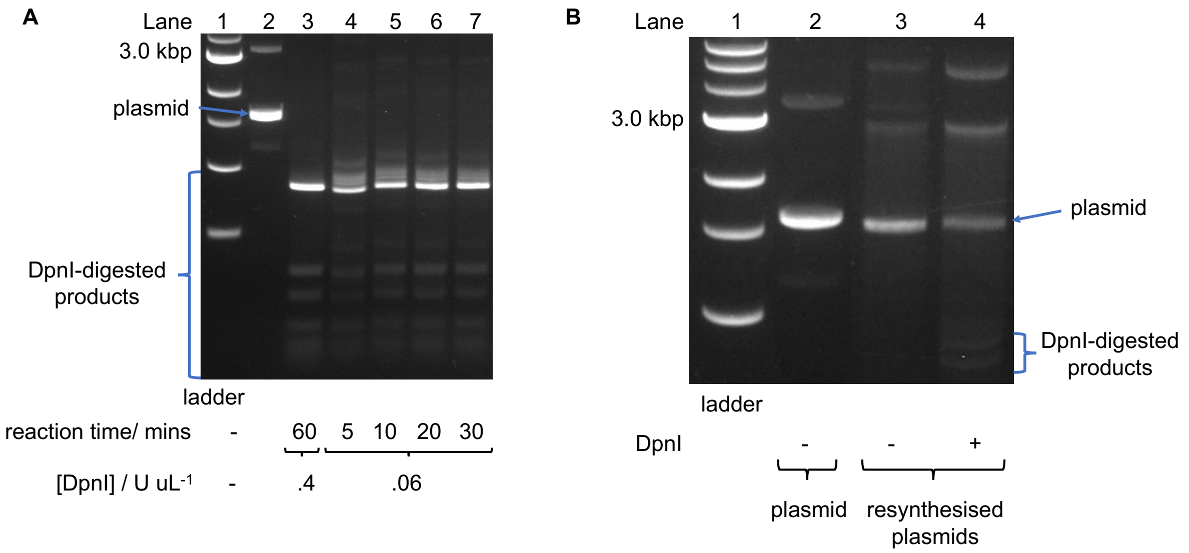

SI Figure 3. DpnI removal of unreacted plasmids. A. Mild DpnI conditions were optimised with native mVenus plasmid. **B.** When resynthesised plasmids were reacted with DpnI faint degraded plasmid bands appeared whilst the majority of the resynthesised plasmids were intact.

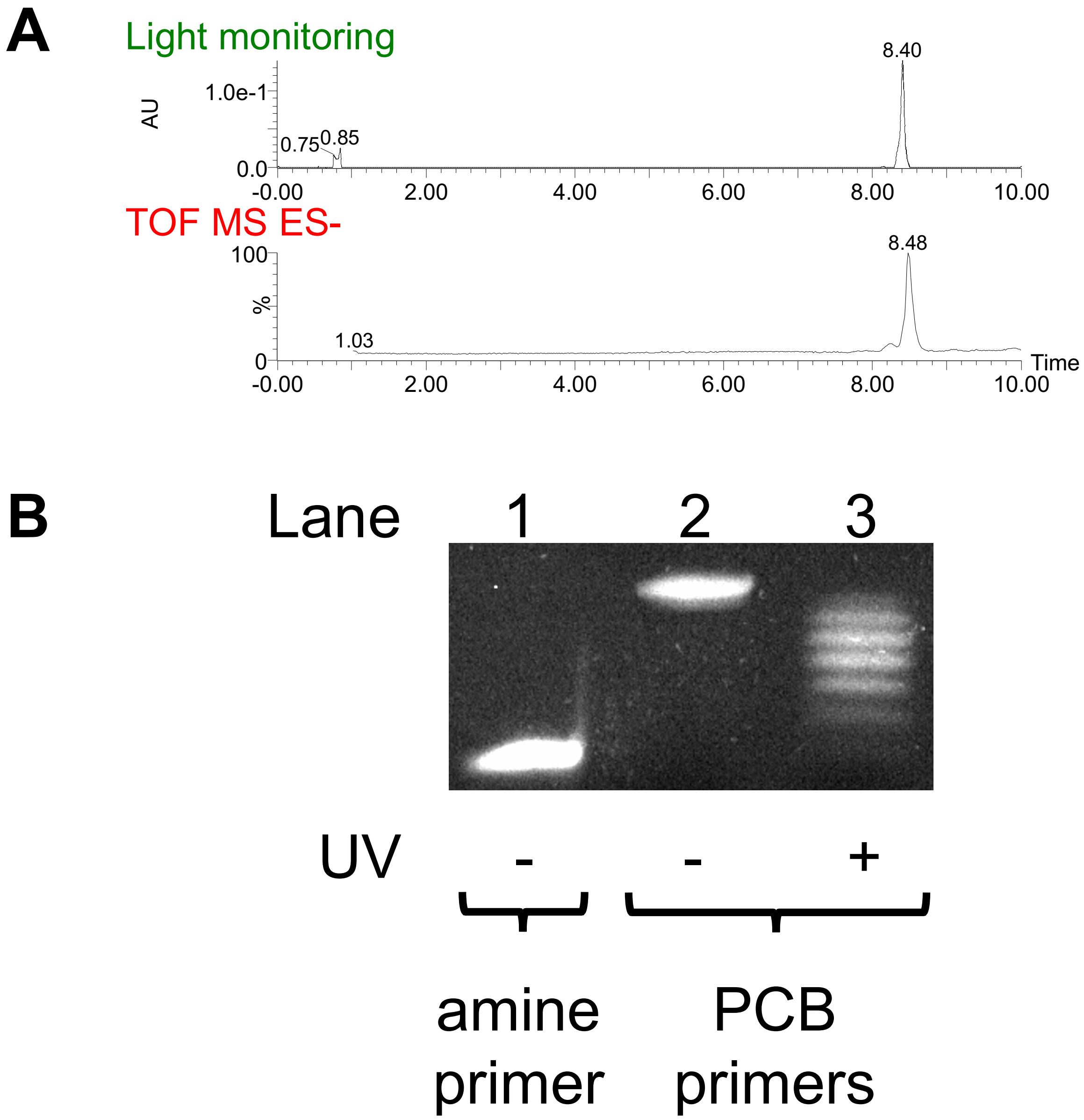

SI Figure 4. Confirming synthesis of the T7 PCB oligo. A. LC-MS and **B.** Denaturing PAGE were used to confirm PCB attachment. MS gave a mass of 15398, which corresponded to the 7-PCBs oligo.

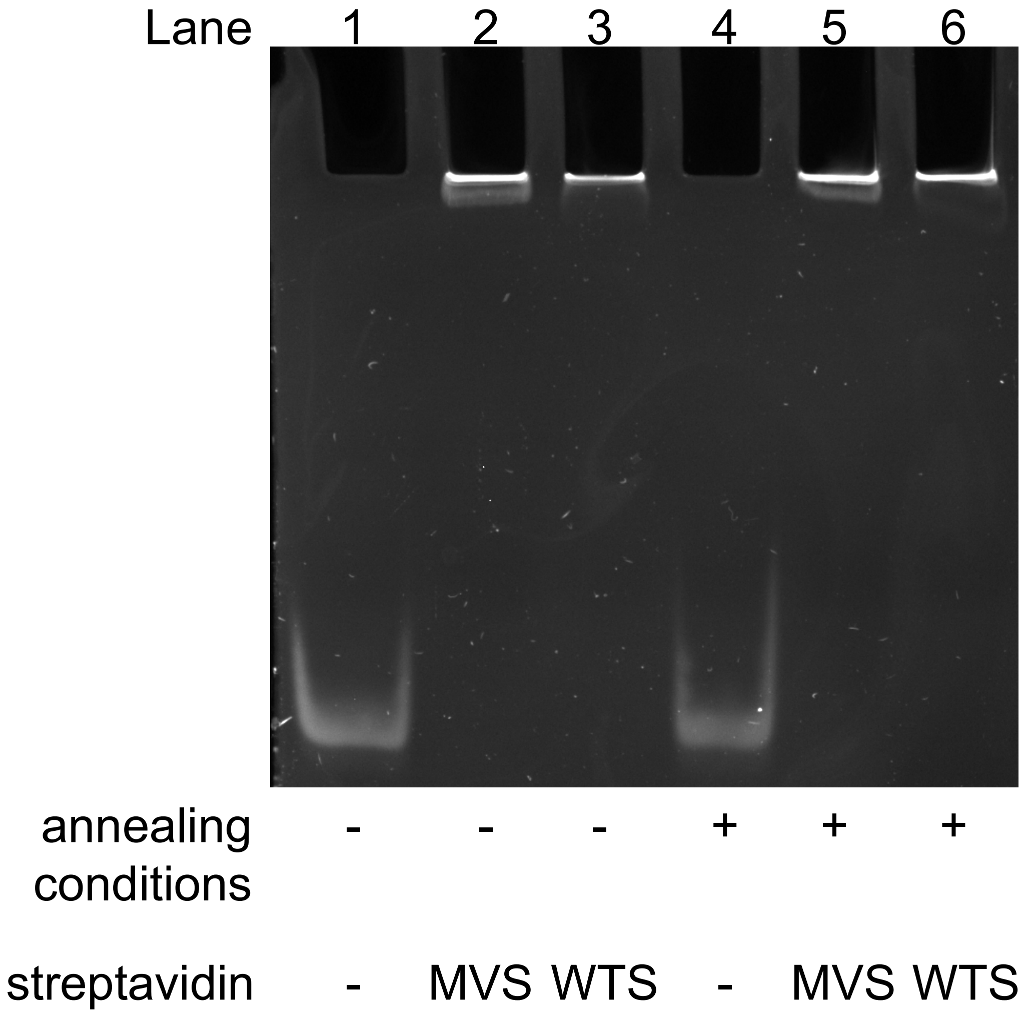

SI Figure 5. PCB oligo was unaffected by annealing conditions. PCB oligo was bound to either monovalent or wildtype tetravalent streptavidin (MVS and WTS, respectively) and incubated in annealing conditions.

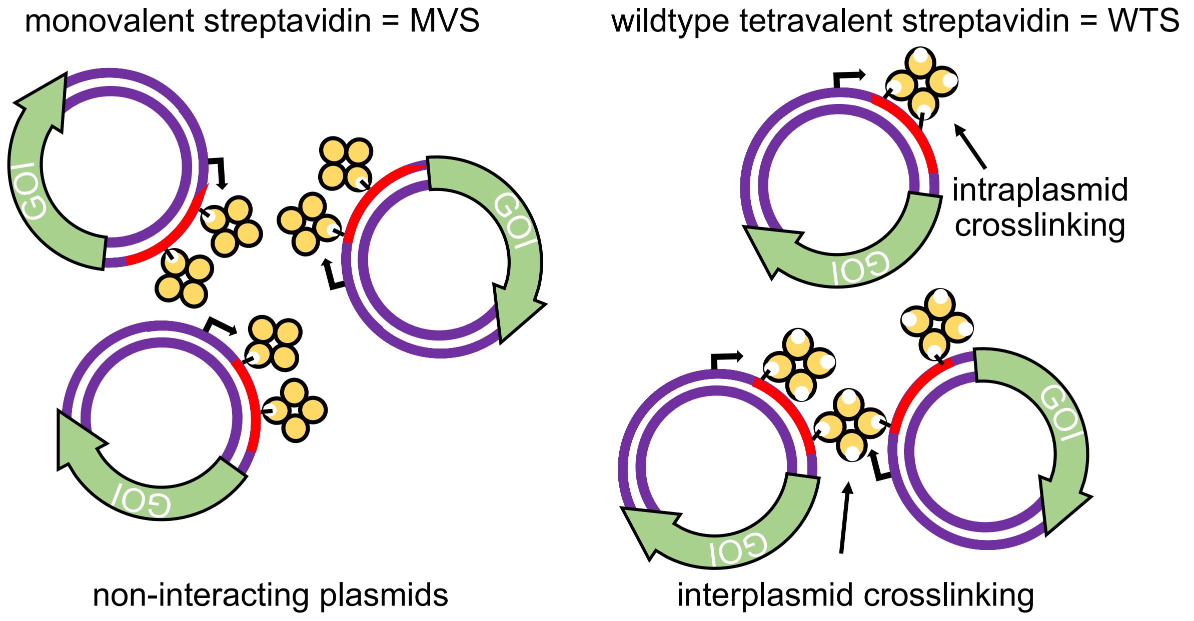

SI Figure 6. Comparison between MVS and WTS LA-plasmids. With only a single active subunit, MVS LA-plasmids do not interact. WTS has four active subunits, which may give rise to intra- and interplasmid crosslinking.

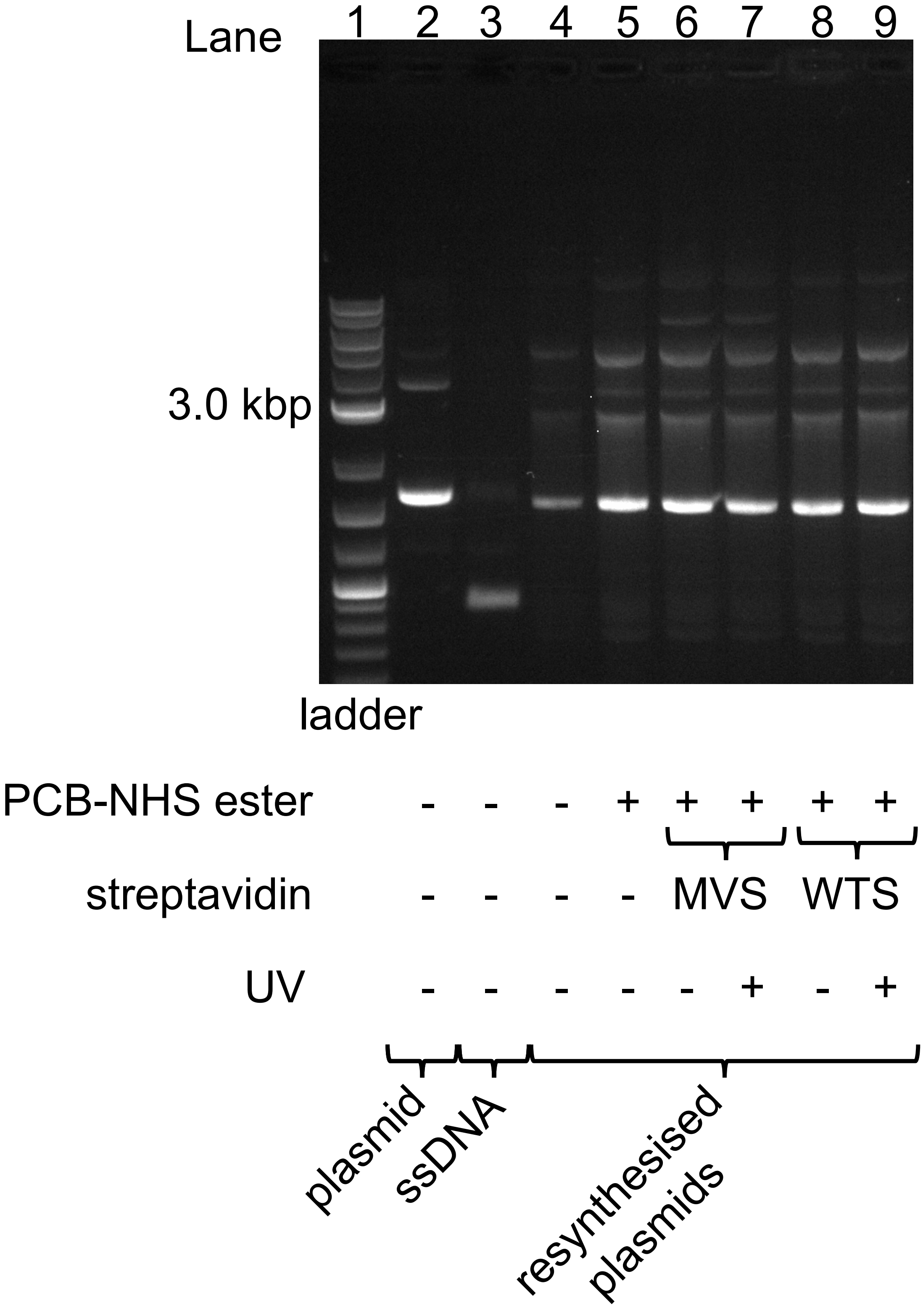

SI Figure 7. Negative controls without amino modifications. Plasmids were resynthesised using a regular primer without amino modifications. The absence of primary amine modifications prevented PCB conjugation and thereby streptavidin binding.

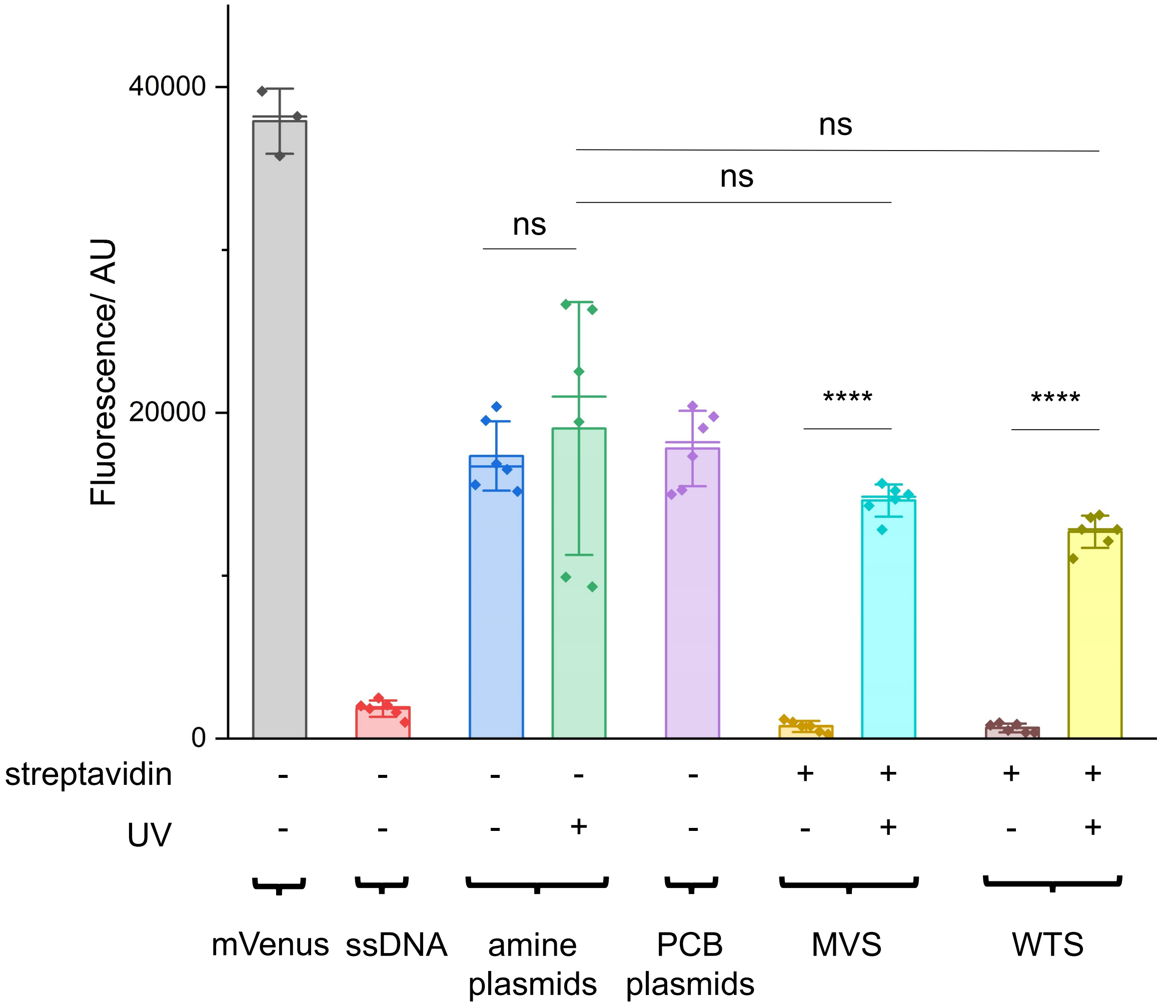

SI Figure 8. CFPS of T7 NEEL plasmids including the native plasmid. Plasmids containing the T7 promoter were prepared with NEEL and assessed using CFPS. Native plasmid and NTC were technical triplicates (n = 3). Replicates, measurements, and statistical analysis were similar to those in **Figure 4B**.

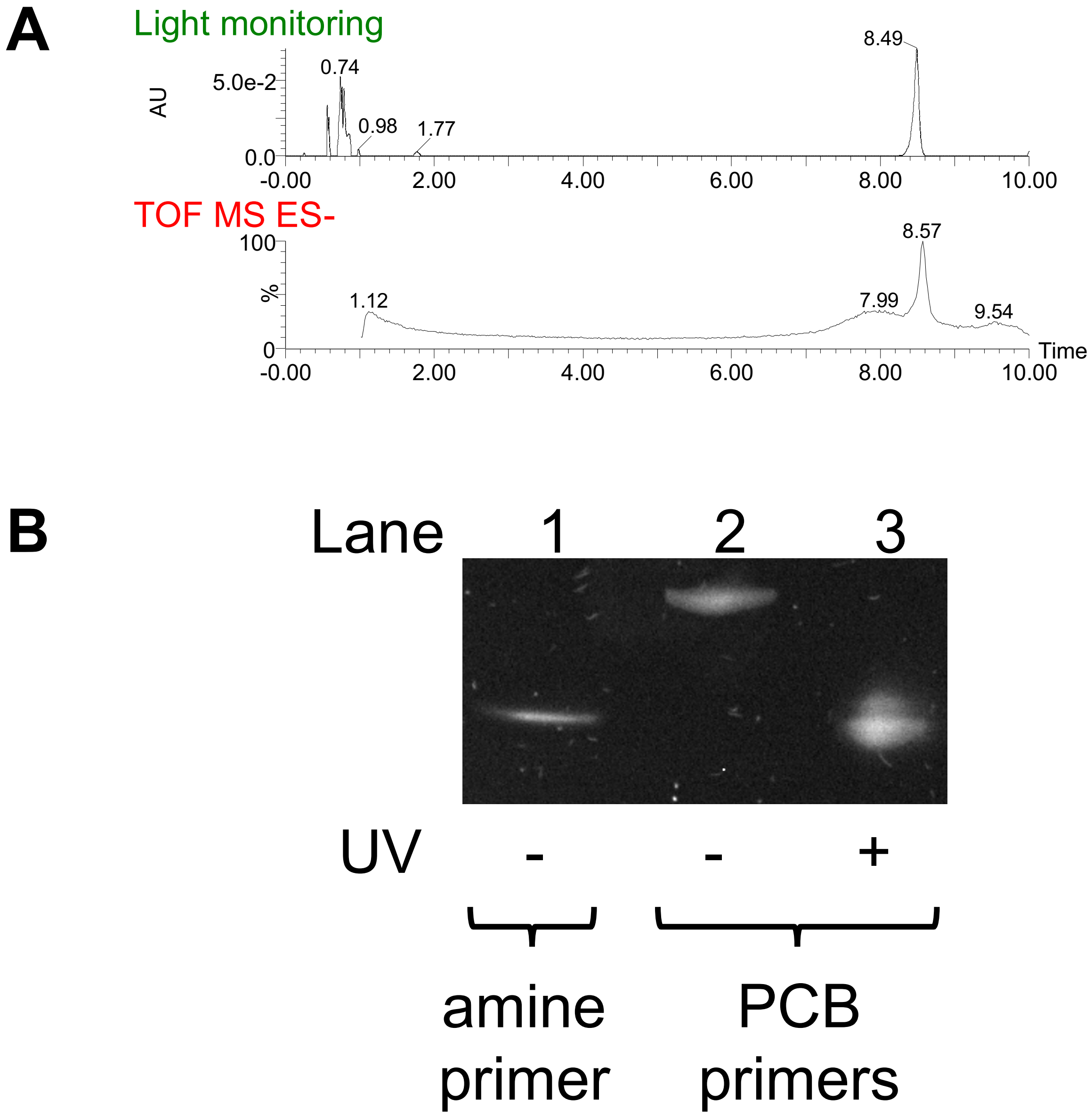

SI Figure 9. Confirming synthesis of the CMV PCB oligo A. LC-MS and **B.** Denaturing PAGE were used to confirm PCB attachment. MS gave a mass of 22076, which corresponded to the 9-PCBs oligo.

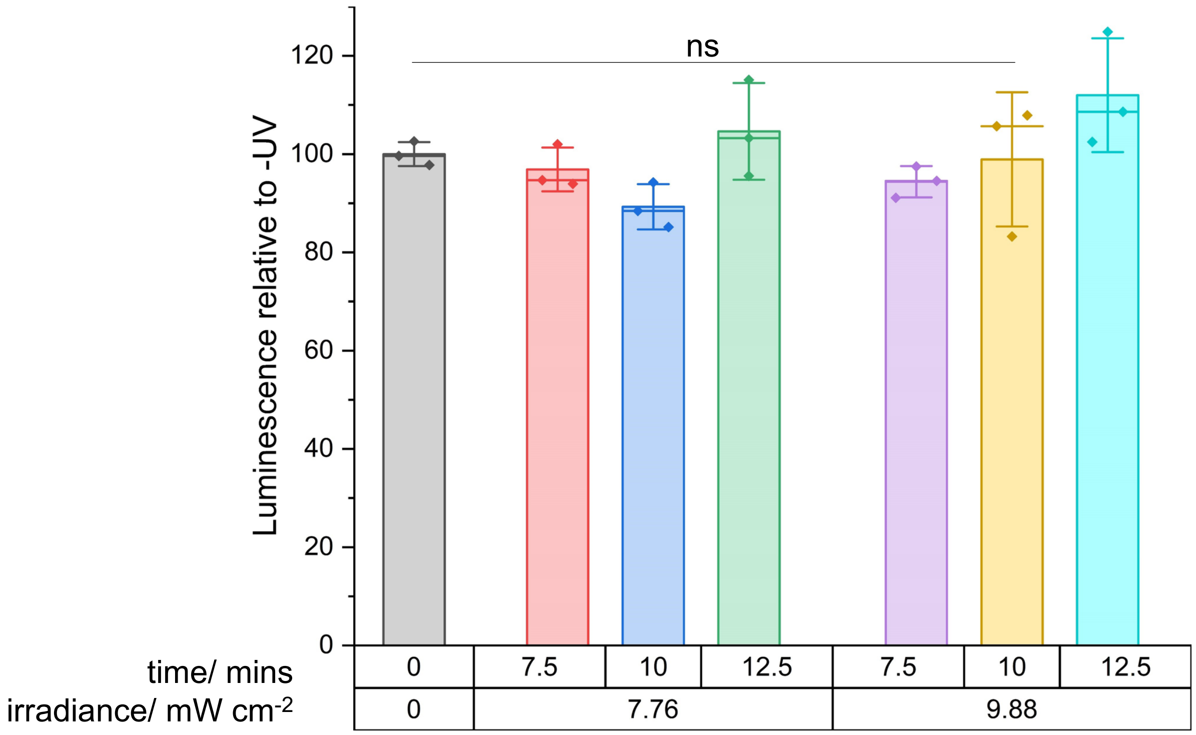

SI Figure 10. Cell viability assay with optimised UV settings for transfection. Cell viability assay with various UV settings. The conditions employed, 9.88 mW cm^{-2} for 10 mins, had minimal damage to cells. Error bars showed standard deviations of technical triplicates ($n = 3$). Two-tailed, unpaired Student's t-test was performed. ns = non-significant ($P > .05$).

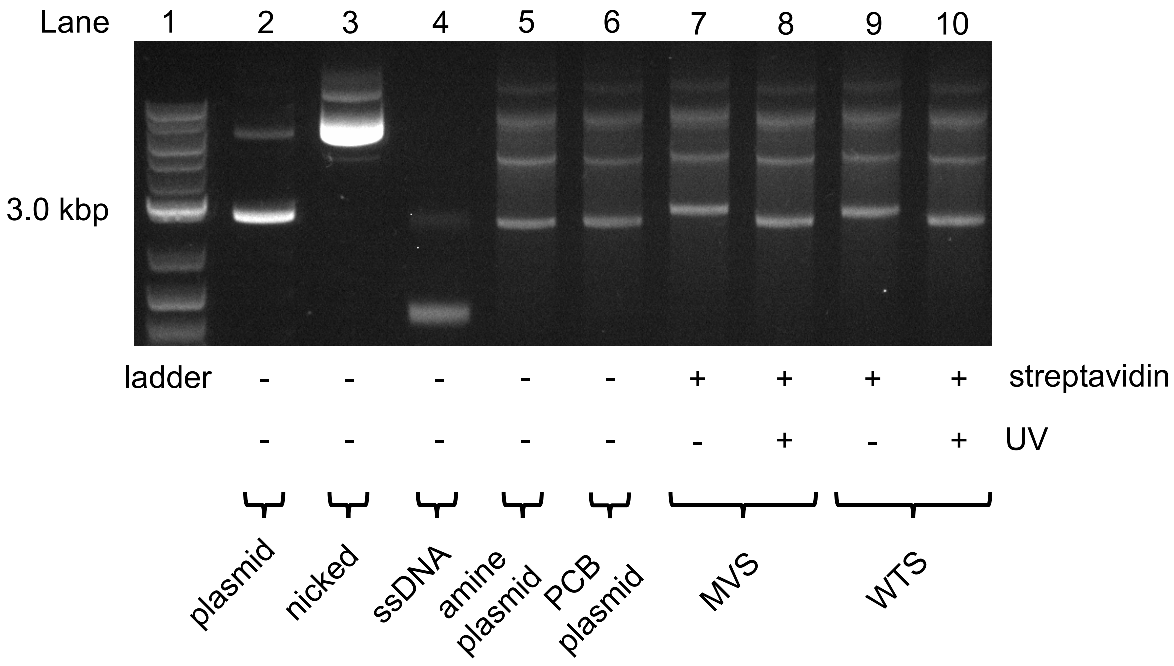

SI Figure 11. CMV LA-plasmids and their uncaging. The PCB plasmids were bound to streptavidins and uncaged using the optimised UV settings in **SI Figure 10**.

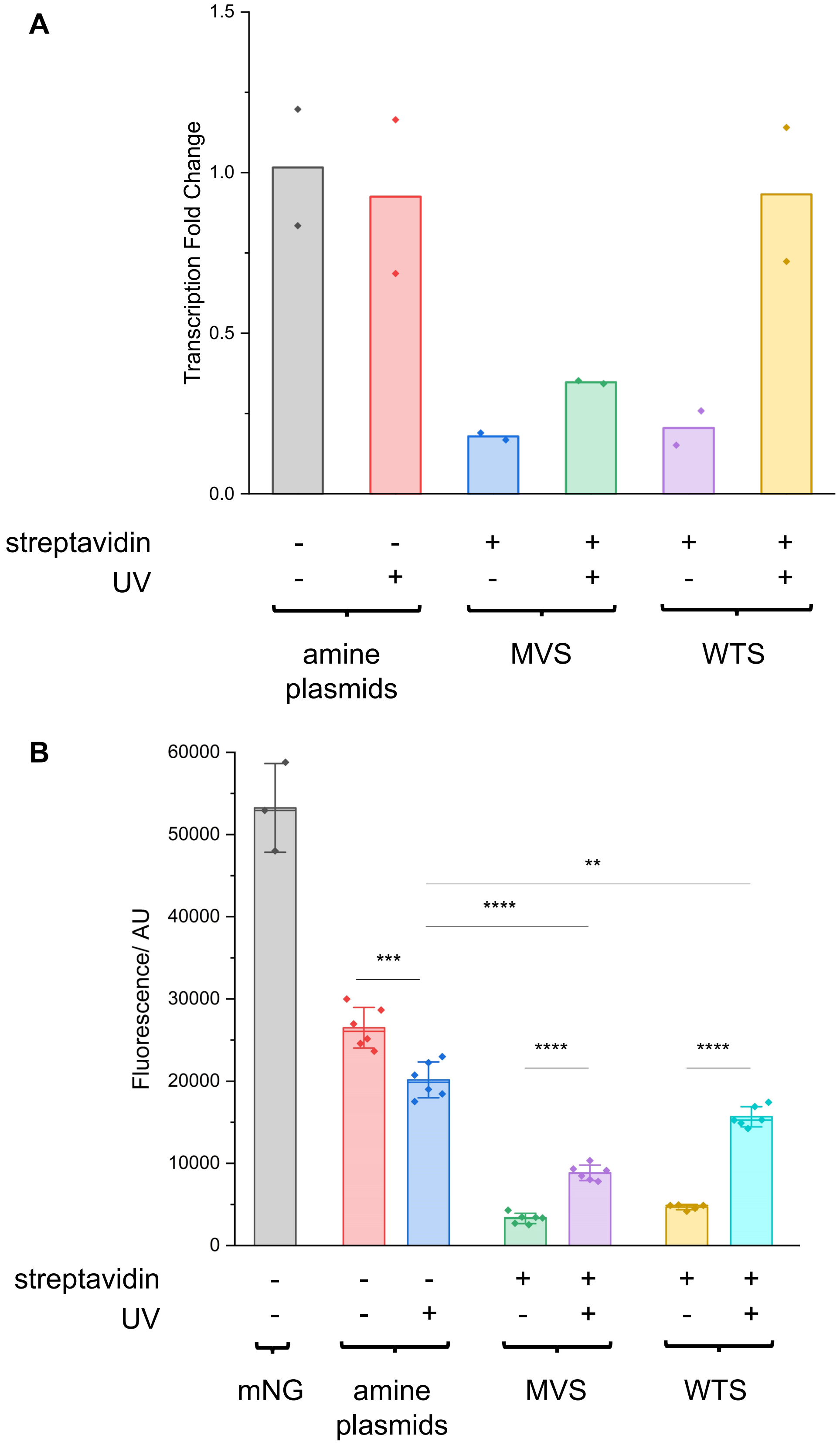

SI Figure 12. RT-qPCR and fluorescence measurements of CMV NEEL plasmids, including the native plasmid. Plasmids containing the CMV promoter were prepared with NEEL and assessed using **A.** RT-qPCR and **B.** Fluorescent plate reader. Replicates, measurements, and statistical analysis were similar to those in **Figure 5**.

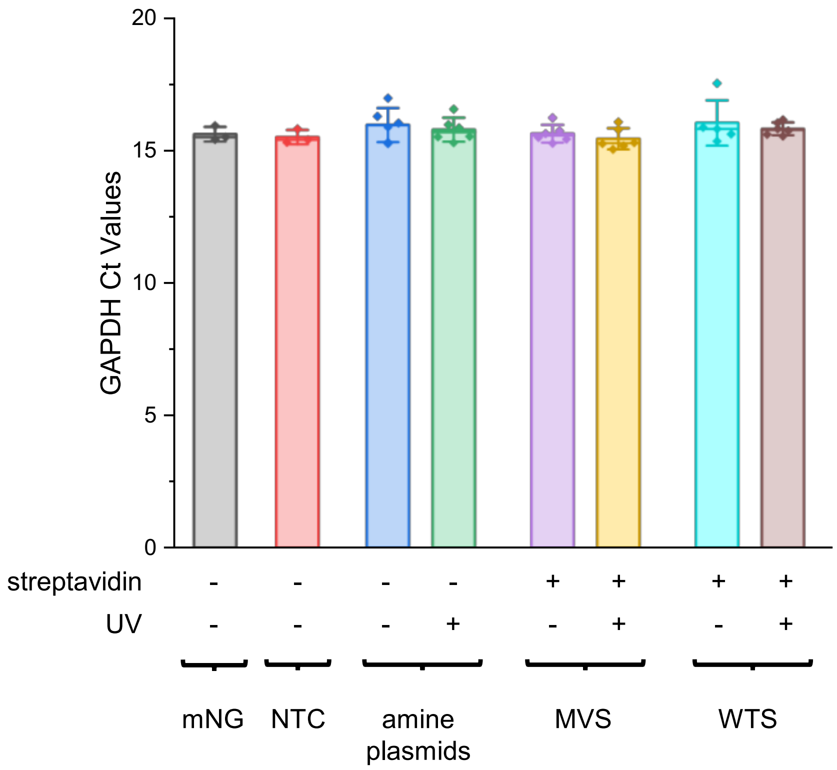

SI Figure 13. GAPDH transcription levels after transfection with modified plasmids. Plasmids containing the photocaged CMV promoter were transfected into HEK293T cells and irradiated with UV. RT-qPCR was performed and the Ct values of the GAPDH gene were compared. Error bars show standard deviations. Two-tailed, unpaired Student's t-test was performed. ns = non-significant ($P > .05$); **$.001 \leq P \leq .01$; ***$.0001 \leq P \leq .001$ and ****$P \leq .0001$. 'mNG' and 'NTC' conditions were technical triplicates ($n = 3$), and all other conditions were biological duplicates of technical triplicates ($n = 6$) except for the 'WTS-UV' condition ($n = 5$ due to sample loss).

References

1. Karata, K., Vidal, A. E. & Woodgate, R. Construction of a circular single-stranded DNA template containing a defined lesion. *DNA Repair* **8**, 852–856 (2009).
2. Huang, R., Fang, P. & Kay, B. K. Improvements to the Kunkel mutagenesis protocol for constructing primary and secondary phage-display libraries. *Methods* **58**, 10–17 (2012).
3. Tonikian, R., Zhang, Y., Boone, C. & Sidhu, S. S. Identifying specificity profiles for peptide recognition modules from phage-displayed peptide libraries. *Nat Protoc* **2**, 1368–1386 (2007).
4. Handa, P. & Varshney, U. Rapid and reliable site directed mutagenesis using Kunkel's approach. *Indian J Biochem Biophys* **35**, 63–66 (1998).
